# RNA-Interference Components Are Dispensable for Transcriptional Silencing of the *Drosophila* Bithorax-Complex

**DOI:** 10.1371/journal.pone.0065740

**Published:** 2013-06-13

**Authors:** Filippo M. Cernilogar, A. Maxwell Burroughs, Chiara Lanzuolo, Achim Breiling, Axel Imhof, Valerio Orlando

**Affiliations:** 1 Epigenetics and Genome Reprogramming Laboratory, IRCCS Fondazione Santa Lucia, Rome, Italy; 2 Adolf-Butenandt Institute, Ludwig Maximilian University of Munich, Munich, Germany; 3 Division of Epigenetics, DKFZ-ZMBH Alliance, German Cancer Research Center, Heidelberg, Germany; 4 CNR Institute of Neurobiology and Molecular Medicine, IRCCS Santa Lucia Foundation, Rome, Italy; 5 RIKEN Omics Science Center, RIKEN Yokohama Institute, Tsurumi-ku, Yokohama, Kanagawa, Japan; 6 Munich Center of Integrated Protein Science, Ludwig Maximilian University of Munich, Munich, Germany; St Jude Children's Research Hospital, United States of America

## Abstract

**Background:**

Beyond their role in post-transcriptional gene silencing, Dicer and Argonaute, two components of the RNA interference (RNAi) machinery, were shown to be involved in epigenetic regulation of centromeric heterochromatin and transcriptional gene silencing. In particular, RNAi mechanisms appear to play a role in repeat induced silencing and some aspects of Polycomb-mediated gene silencing. However, the functional interplay of RNAi mechanisms and Polycomb group (PcG) pathways at endogenous loci remains to be elucidated.

**Principal Findings:**

Here we show that the endogenous Dicer-2/Argonaute-2 RNAi pathway is dispensable for the PcG mediated silencing of the homeotic Bithorax Complex (*BX-C*). Although Dicer-2 depletion triggers mild transcriptional activation at Polycomb Response Elements (PREs), this does not induce transcriptional changes at PcG-repressed genes. Moreover, Dicer-2 is not needed to maintain global levels of methylation of lysine 27 of histone H3 and does not affect PRE-mediated higher order chromatin structures within the *BX-C*. Finally bioinformatic analysis, comparing published data sets of PcG targets with Argonaute-2-bound small RNAs reveals no enrichment of these small RNAs at promoter regions associated with PcG proteins.

**Conclusions:**

We conclude that the Dicer-2/Argonaute-2 RNAi pathway, despite its role in pairing sensitive gene silencing of transgenes, does not have a role in PcG dependent silencing of major homeotic gene cluster loci in *Drosophila*.

## Introduction

RNA-based silencing mechanisms are a widespread phenomenon in eukaryotes and act at multiple levels to regulate gene expression [1]–[3]. In *Drosophila* small RNA-mediated gene silencing depends on two groups of genes encoding the key proteins of the Dicer and Argonaute families [4], [5]. Dicer-1 (Dcr1) generates microRNAs (miRNAs) whereas Dicer-2 (Dcr2) creates small interfering RNAs (siRNAs). Argonaute (AGO) proteins directly bind small guide RNAs and either display endonucleolytic activity or serve as a platform for the assembly of silencing complexes [6]. The *Drosophila* Argonaute proteins can be divided into two groups: the ubiquitous AGO (AGO1 and AGO2) and the germline-specific Piwi subfamilies [6]. AGO1 is involved in the miRNA dependent pathway that silences messenger RNA, whereas AGO2 functions in RNA interference (RNAi) by exogenous and endogenous siRNAs [7]–[10]. Piwi proteins are involved in transposon silencing and heterochromatin formation [11]–[14].

Accumulating evidence indicates that RNAi components and small RNAs act in the nucleus to control heterochromatin formation, repeat-induced gene silencing and transposable element mobilization [15], [16]. In particular, extensive data from fission yeast suggest that bidirectional transcription from repetitive DNA-sequences creates dsRNA molecules that are cleaved by the Dicer enzyme into siRNAs of 21–23 nucleotides of length. These siRNAs are employed to guide the RNA-induced transcriptional silencing (RITS) complex to homologous sequences in the genome [17], [18]. This leads to the recruitment of histone methyl transferases, methylation of lysine 9 of histone H3, which promotes the formation of heterochromatin via the recruitment of HP1-like proteins [19]. Although the studies in fission yeast represented a paradigm for how RNAi would control gene expression at the chromatin level the simple transposition of this model to other organisms is not always applicable. We have recently shown that in *Drosophila* RNAi components act in the nucleus, preferentially associate with transcriptionally active loci and control RNA polymerase II processivity [20]. However, based also on other reports, the link between the RNAi pathway, heterochromatin formation and related aspects of epigenetic gene silencing in *Drosophila* remains unclear [21]–[23].

The close relationship with heterochromatin inspired also research regarding a potential link between Polycomb group (PcG) proteins and RNAi. PcG proteins convey epigenetic inheritance of repressed transcriptional states through several rounds of cell division by regulating multiple levels of chromatin structure [24], [25]. They act as large multi-protein complexes grouped into the PRC1 and PRC2 (Polycomb Repressive Complex 1 and 2, respectively) subgroups, preventing changes in early-determined transcriptional repressive states of developmentally-regulated genes. The *Drosophila* PRC2 complex contains the intrinsic Histone Methyl Trasnsferase (HMTase) Enhancer of zeste (E(z)), that methylates preferentially lysine 27 of histone H3 (H3K27), which in turn recruits the Polycomb protein (PC) that is a stoichiometric component of PRC1 [26]–[28].

Most studies on PcG focused on the well known *Drosophila* Bithorax Complex (*BX-C*). This locus contains three coding genes *Ultrabithorax* (*Ubx*), *abdominal A* (*abd-A*) and *Abdominal B* (*Abd-B*). In *Drosophila*, PcG function is mediated by specialised modular DNA elements called Polycomb Response Elements (PREs) that, together with core promoters, assemble in characteristic multilooped structures necessary for the maintenance of PcG mediated silencing [29]–[33].

A possible role for RNAi components in PcG mediated gene silencing has been reported [34]. In *Tetrahymena* EZL1, homolog of E(z), is responsible for H3K27 methylation in a RNAi-dependent manner [35], while in human cells RNAi-mediated transcriptional gene silencing requires the mammalian E(z)-homologue EZH2 [36]. In *Drosophila* PcG-dependent transcriptional silencing of multiple copies of transgenes (co-suppression) involves the RNAi-machinery [37], [38]. Similarly, it was shown that RNAi-components are required for pairing sensitive gene silencing controlled by PREs [39]. Of note, these forms of silencing seem to depend on direct interactions between transgenic PRE-sequences accompanied by the production of RNAs of 21–22 nucleotides in length from transgenic PREs, while no 21–22 nt RNAs could be detected from endogenous PREs. Only some PcG proteins are displaced from the affected transgene in RNAi mutants [39]. In addition, although mutations in RNAi components perturb the nuclear clustering of endogenous Polycomb repressed *HOX* loci [39], RNAse treatment has no effect on the higher order structures of *HOX* clusters [33].

Further, a recent report including high resolution mapping of AGO2 by chromatin immunoprecipitaion followed by high throughput sequencing (ChIP-seq) and systematic genetic analyses could not detect a direct link between RNAi and PcG pathways [40]. Thus the involvement of RNAi machinery components in gene silencing at endogenous PcG target loci remains unclear [34].

Here we show that the Dcr2/AGO2 pathway is not required to maintain global levels of H3K27 methylation, transcriptional silencing of the *BX-C* homeotic genes and PRE-specific noncoding RNAs. In addition Chromosomal Conformation Capture (3C) analysis showed that higher order structures of the *BX-C* do not depend on Dcr2. By comparing published data sets of PcG targets with AGO2-interacting small RNAs (sRNAs) we observe no enrichment of AGO2-sRNAs at promoter regions associated with PcG proteins. We conclude that RNAi-related mechanisms are largely not involved in gene silencing at endogenous PcG target loci in *Drosophila.*

## Results

### PRE-specific Transcripts as Potential Sources of dsRNAs in the *BX-C*

**Figure 1:**
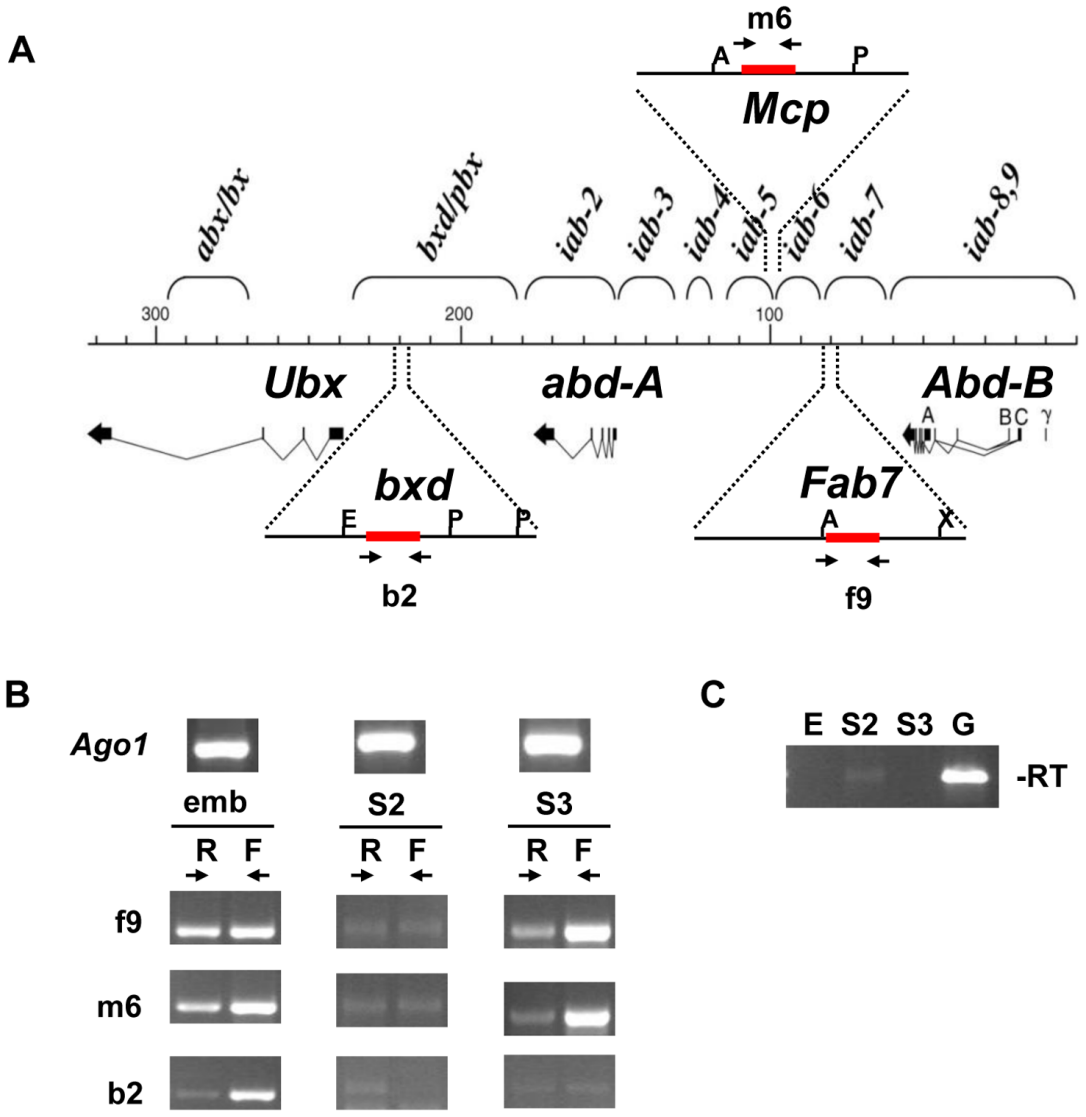
Potential sources of dsRNA at PREs. (**A**) Schematic representation of the *BX-C*. *Fab-7, Mcp* and *bxd* regions are highlighted and the positions of the primers used in this study are indicated by arrows. All amplicons are about 500 bp long. Core PRE regions are highlighted in red. A: ApaI restriction site (*Fab-7*) or AccI site (*Mcp*); E: EcoRI site; P: PstI site; X: XbaI. (**B**) Analysis of strand specific transcripts at *BX-C* PREs. RT-PCR was performed on total RNA (DNAse treated) from embryos (0–18 h after egg laying), S2 and S3 cells. Primers complementary to forward (F) or reverse (R) strand transcripts (the homeotic genes of the *BX-C* are transcribed from the forward strand) were used to initiate cDNA synthesis; subsequently, the other primer was added for PCR amplification. As input control a primer pair specific for transcripts of the *Argonaute-1 (Ago1)* gene was included (top). (**C**) To check for genomic contamination total RNA was PCR amplified using primers f9 without the RT step (-RT). As positive control genomic (G) DNA was also amplified.

In order to assess the role of the RNAi machinery in silencing of homeotic gene clusters in *Drosophila melanogaster* we analyzed the presence of putative dsRNAs at *BX-C* PREs. It is known that the transcription of non-coding RNAs through PREs correlates with the maintenance of the active state of homeotic PcG-targets in the *BX-C* [34], [41]–[43]. We thus performed strand specific RT-PCR analysis in the *Fab-7*, *Mcp* and *bxd* PREs by using the primer pairs indicated in Fig. 1A. Using cDNA from embryos, we found that the *Fab-7*, *Mcp* and *bxd* core PREs produce transcripts from both strands (primers f9, m6 and b2, respectively; Fig. 1B). Next we compared *Drosophila* S2 and S3 cultured cells. In the S2 and S3 cells used in this study *Ubx* and *abd-A* are repressed and transcribed at low levels; in contrast the three transcripts of *Abd-B* are strongly transcribed in S3 but not in S2 cells [33], [44] **.** In the *bxd* region, the PRE controlling *Ubx* expression, no transcripts were detected in either S2 or S3 cells (Fig. 1B). Conversely, transcripts from both strands were detected in S3 but not in S2 cells in the core regions of *Mcp* and *Fab-7*, which are the PREs that control *Abd-B* expression (Fig. 1B). Thus, transcription through the *Mcp* and *Fab-7* PREs appears to correlate positively with the expression of *Abd-B*, which is in agreement with previous findings [41]–[43].

We then hypothesized that a potential role of the RNAi machinery in PcG repression could be to rapidly process PRE-specific transcripts by the Dcr2 enzyme in regions, where the corresponding *BX-C* gene is inactive. These small RNAs (sRNAs) should then be loaded in the AGO2 RNAi effector complex. To test this hypothesis we analyzed our recently published data set of AGO2-bound small RNAs [20] to map potential matching sites in the *BX-C* region. Briefly, the sequenced sRNA libraries were generated from RNA fractions obtained from S2 cells by immunoprecipitation with antibodies against AGO2 or control IgG. The entire *BX-C* genomic region was scanned for overlaps with sRNA sequences and no enrichment was detected (Table 1). We then ran 10,000 simulations randomly selecting *Drosophila* genome regions of equivalent size to the *BX-C* (∼340 kb) and summed the sRNA counts for these regions (Fig. S1). The total AGO2-bound sRNA counts for the *BX-*C are consistent with counts observed from random ∼340 kb genomic regions, suggesting the *BX-C* is not enriched for sRNAs associated with AGO2. In addition we performed RNAse protection analysis with different sense and antisense probes to scan *Fab-7* and *bxd* endogenous PRE regions for the presence of homologous sRNAs. Representative pictures are shown in Fig. S2. Since in this analysis we used sRNA preparations of molecules shorter than 200 nt we could not detect the PRE transcripts shown in Fig. 1B. In all cases we did not observe RNA molecules matching the typical 21–23 nucleotide length of a Dcr2 product. sRNAs of 10 nucleotides detected represent most likely technical artifacts (Fig. S2A, B), that are present also in embryos carrying a *Fab-7* PRE deletion (Fig. S2C). To test our assay system, we screened for the miRNA *bantam* that is known to be expressed in S2 cells [45]. As shown in Fig. S2D, *bantam* was readily detected in our pool of S2-specific sRNAs. These results confirm the absence of siRNAs of 21–23 nucleotides from endogenous PcG regulated loci [39].

**Table 1:** AGO2-bound sRNAs tags mapping in the *BX-C*.

IPs	raw tags	normalized tpm	fold change
**AGO2-IP**	146743.3	12298.66	1.12
**neg.-IP**	50100.79	10943.09	

AGO2-bound sRNAs tags [20] were analyzed for their mapping in the *BX-C* region. TPM: tags per million; fold change: calculated as ratio AGO2-IP/negative-IP normalized tpm.

**Figure 2:**
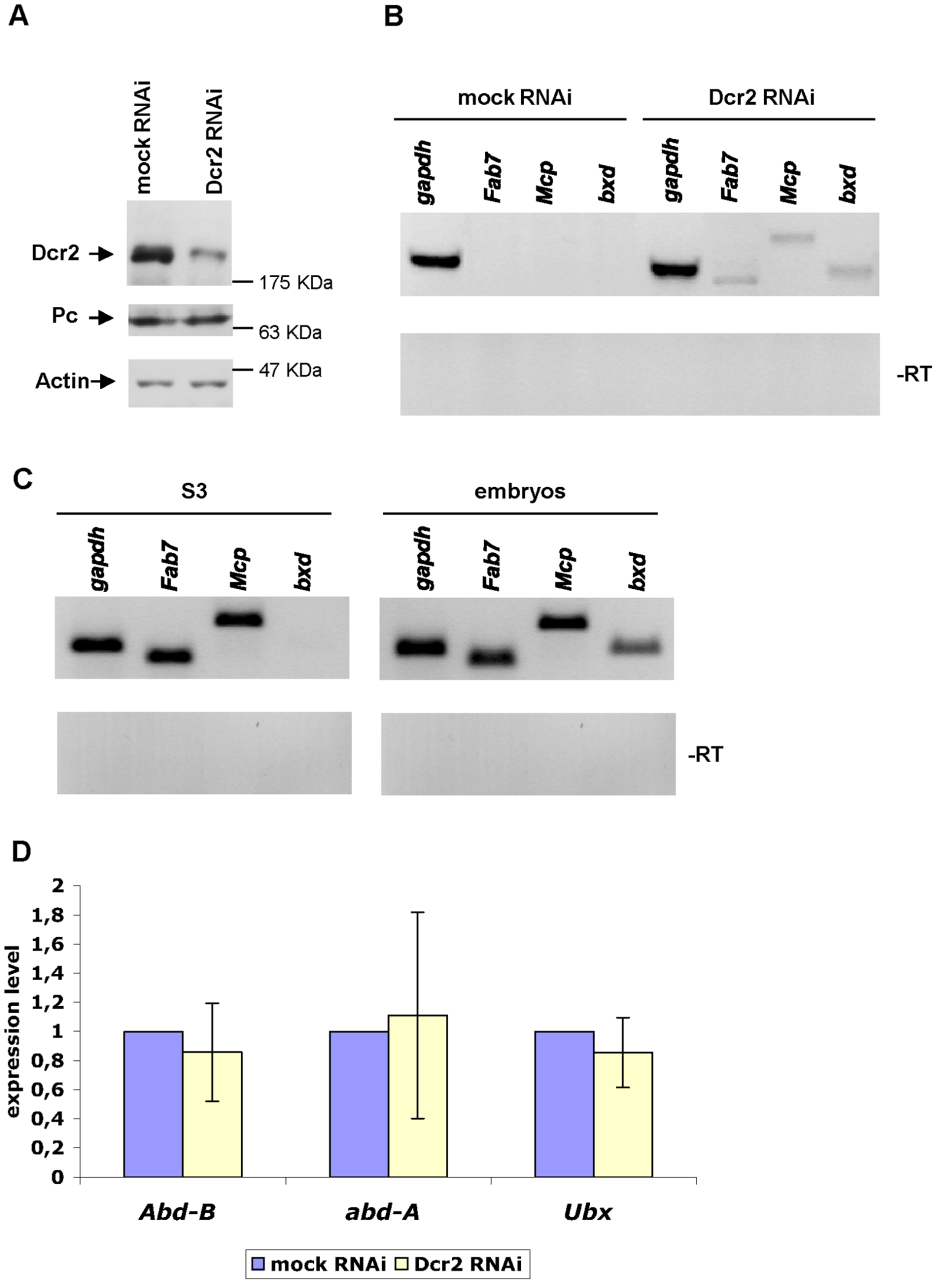
Dcr2 depletion does not cause transcriptional activation of homeotic genes. (**A, B**) The samples analyzed were mock-treated S2 cells (control transfection without dsRNA) or S2 cells treated with Dcr2 dsRNA. **(A)** Western blot with the indicated antibodies. (**B**) RT-PCR analysis of *glyceraldehyde-3-phosphate dehydrogenase* (*gapdh*) and core PRE transcripts. To check for genomic DNA contamination total RNA from each sample was PCR amplified without the RT step (-RT). (**C**) RT-PCR analysis in S3 cells and embryos (0–18 h after egg laying). To check for genomic DNA contamination total RNA from each source was PCR amplified without the RT step (-RT). (**D**) Quantitative RT-PCR of the *BX-C* homeotic genes. The samples analyzed were mock-treated S2 cells (control without dsRNA) or S2 cells treated with Dcr2 dsRNA. The results shown are from three independent experiments; error bars show the standard deviation.

In order to further analyse whether Dcr2 has a role in regulating PRE-transcripts and by that the expression of *BX-C* genes, we next depleted Dcr2 in S2 cells. As shown in Fig. 2A, we observed a specific reduction of the Dcr2 protein level, while the PC protein was not affected. Notably, Dcr2 depletion in S2 cells has been shown to also reduce AGO2 protein levels, suggesting that Dcr2 and AGO2 stabilize each other in a protein complex. [20]. PRE-specific transcripts became detectable upon Dcr2 knock down (Fig. 2B, S3A), though the abundance of these transcripts is largely below the amounts found in S3 cells and embryos (Fig. 2C, S3B). Moreover, we did not observe any significant de-repression of homeotic *BX-C* genes in cells depleted for Dcr2 (Fig. 2D, S3C). Thus, Dcr2 depletion has only a moderate effect on PRE-specific transcripts and does not cause loss of silencing at homeotic *BX-C* genes.

### Consequences of Dcr2 Depletion on Chromatin Marks at PREs

**Figure 3:**
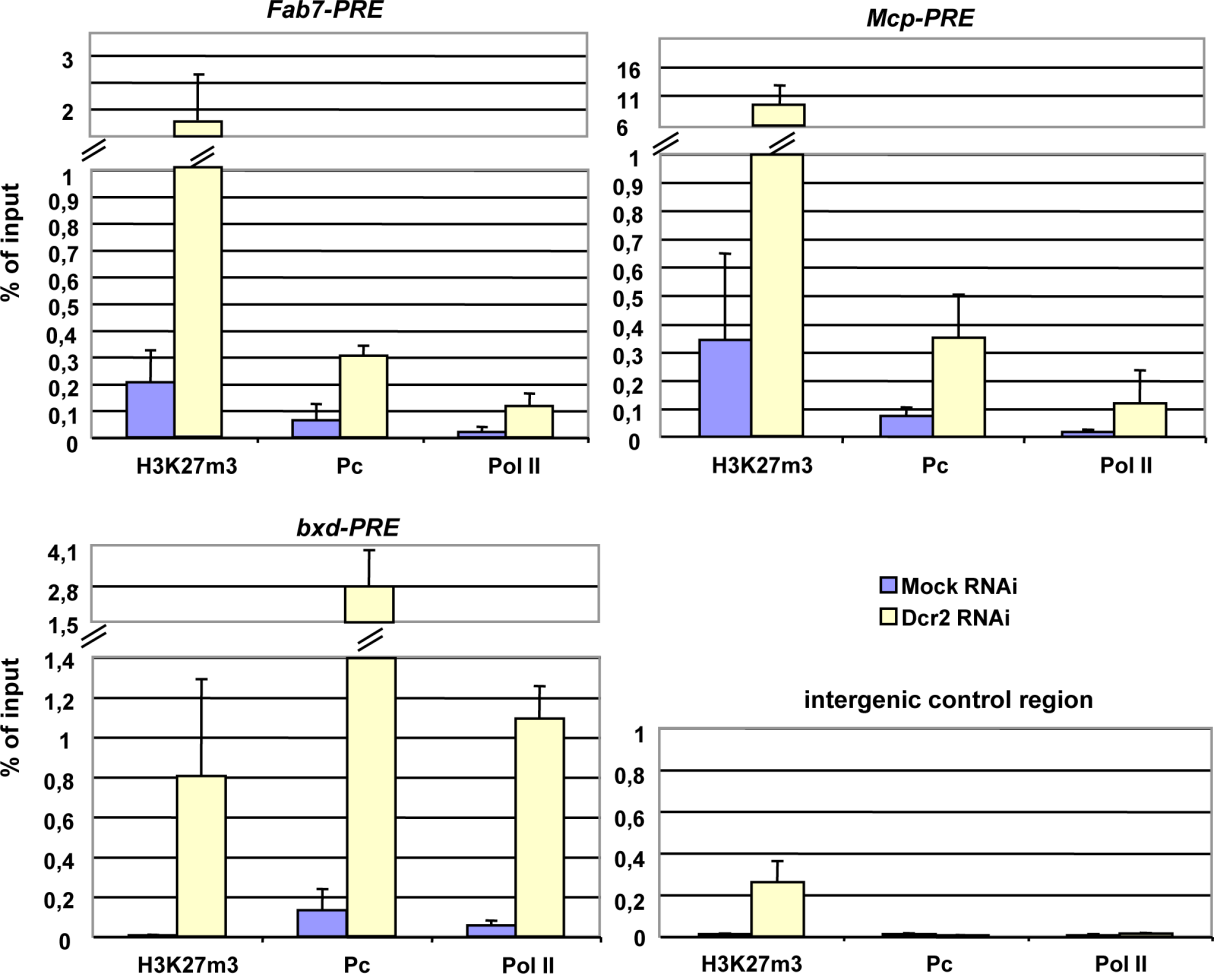
Consequences of Dcr2 depletion on chromatin marks at PREs. Crosslinked chromatin from mock-treated S2 cells (control transfection without the dsRNA) or S2 cells treated with Dcr2 dsRNA was immunoprecipitated with the antibodies indicated below the bars (H3K27m3, PC, Pol II). The immunoprecipitated DNA was analyzed by quantitative PCR with primers specific for the *Fab-7*, *Mcp* and *bxd* core PRE and for an intergenic control region (3,2 kb upstream of CG14356 [62]). Protein binding is expressed as the percentage of input minus the background signal. The results shown are from three independent experiments; error bars show the standard deviation.

We next asked if the moderate increase in PRE transcripts observed in Dcr2 depleted cells was accompanied by reduced levels of PC and/or H3K27 methylation on chromatin, as could be observed upon knock down of PcG-components [44]. Chromatin from control S2 cells and cells depleted for Dcr2 was used for immunoprecipitations with antisera against H3K27m3, PC and RNA Polymerase II (Pol II). In agreement with mild up-regulation of PRE-transcripts (Fig. 2B) we observed an increase of Pol II in Dcr2 depleted cells in all three PRE cores sites analyzed, but not in the intergenic control region (Fig. 3). Unexpectedly, the levels of PC and H3K27m3 also increased specifically on PREs, whereas H3K27m3 was found enriched also on the intergenic control region. In addition we analyzed another PcG target region in the promoter of the *Abd-B* gene [44] whose expression level is not altered upon Dcr2 depletion (Fig. 2D). Differently from the PRE cores, we observed an increase only in H3K27m3 (Fig. S4). These results indicate that the moderate transcriptional activation of PREs detected in Dcr2 depleted cells is not accompanied by a loss of PcG proteins, suggesting that Dcr2 function is not required for PcG recruitment and the maintenance of the H3K27m3 mark at PREs.

### Contribution of RNAi to Global Levels of Histone H3K27 Methylation

**Figure 4:**
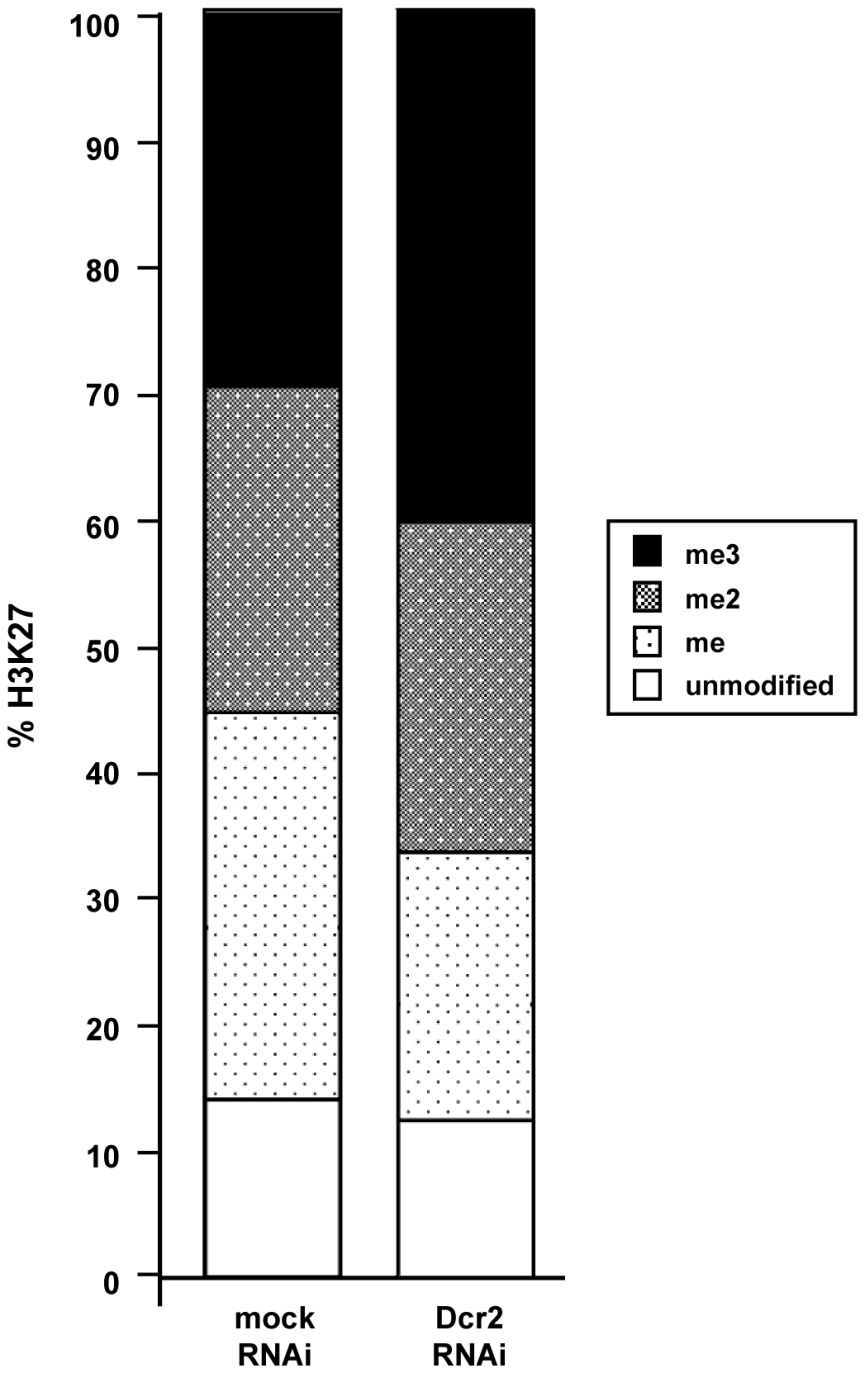
Contribution of the RNAi pathway to H3K27 methylation. Histones were acid extracted from mock treated S2 cells (control transfection without the dsRNA) or S2 cells treated with Dcr2 dsRNA, separated by SDS-PAGE, isolated from the gel and analyzed by MALDI-TOF mass spectrometry. The graph shows the amount of methylation within the peptide containing aminoacids 27–40 of H3 from two independent experiments.

In *Drosophila*, loss of E(z) function results in a loss of monomethylation, dimethylation and trimethylation of H3K27 and a consequent increase of unmodified H3K27, indicating that E(z) is, so far, the only H3K27-specific methyltransferase active in flies [46]. In order to evaluate the general contribution of the RNAi pathway to the maintenance of global H3K27 methylation, histones of control S2 cells and Dcr2 depleted cells were purified and subjected to quantitative *MALDI*-*TOF* mass spectrometry. As shown in figure 4, the peptide containing aminoacids 27–40 of H3 was analysed. This peptide contains three lysines, two of which (K27 and K36) are frequently methylated in vivo. Tandem mass spectrometry (MS/MS) studies using S2 cells showed that the di- and trimethylated forms are predominantly methylated at K27 [47]. Although we observed a slight increase in trimethylation after Dcr2-depletion, in line with the increase of H3K27m3 signals observed upon Dcr2-knock-down in our ChIP experiments (Fig. 3), the overall levels of H3K27 methylation were not perturbed. This analysis shows that Dcr2-dependent pathways are not needed for the maintenance of global levels of H3K27 methylation.

### Dcr2 depletion does not Affect PcG Mediated Higher Ordered Structures in the *BX-C*

**Figure 5:**
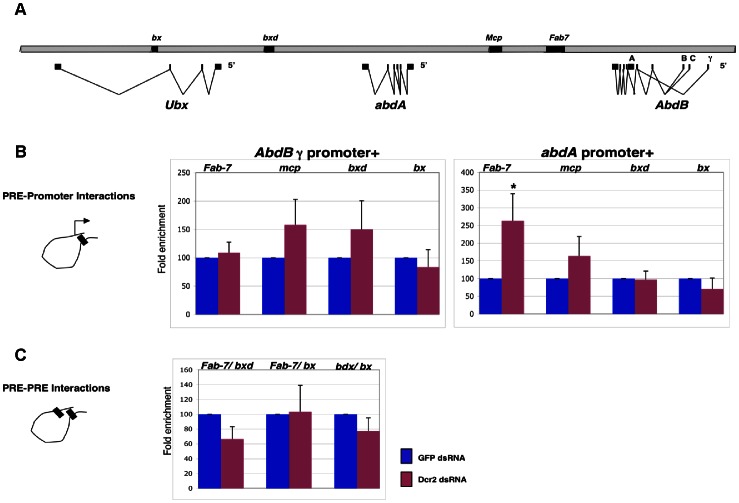
*BX-C* higher order interactions are not impaired after Dcr2 depletion. (**A**) The scheme shows the Bithorax Complex (*BX-C*), including transcription units and genetically characterized regulatory regions (black). (**B–C**) Chromosome Conformation Capture (3C) on S2 cells. Cells were treated with EGFP-dsRNA (control) and Dcr2-dsRNA. Crosslinking frequencies between PcG targets are shown. All data points were generated from an average of at least three independent experiments. The standard error of the mean is indicated. Two-tailed *t*-test was applied for statistical analysis. Asterisks indicate statistically relevant differences; α = 0.05. (**B**) Crosslinking frequencies, normalized on the control, between the fixed fragments spanning homeotic promoters (*Abd-Bγ*; *abd-A*) and *BX-C* PREs. (**C**) PRE/PRE crosslinking frequencies, normalized on the control. Standard error of the mean is indicated.

As mentioned above, it was reported that some components of the RNAi machinery are involved in PcG-mediated pairing-sensitive silencing (PSS) at transgenic *Fab-7* elements. In addition, Dcr2 and other RNAi related proteins influence the nuclear organization of repressed *HOX* loci [39]. We have previously demonstrated by Chromosome Conformation Capture (3C) and fluorescent *in situ* hybridization (FISH), that in the repressed state, all major PcG bound *BX-C* elements, including PREs and core promoters (Fig. 5A), interact at a distance, giving rise to a topologically complex structure dynamically regulated during the cell cycle [33], [48]. These functional interactions are dependent on PcG proteins [49] and are necessary for the maintenance of the transcriptional state of the homeotic genes [30]. In order to examine if these interactions depend on the RNAi machinery, we monitored PRE/promoter (Fig. 5B) and PRE/PRE interactions (Fig. 5C) by 3C in the absence of Dcr2. After Dcr2-dsRNA treatment, the major associations previously observed between regulatory elements in *BX-C* were not impaired (Fig. 5B, C). Notably the frequency of interaction between the *abd-A* promoter and the *Fab-7* PRE was enhanced after Dcr2 depletion (Fig. 5B), although to date, no specific functional role of the *Fab-7* PRE in the regulation of the *abd-A* promoter has been reported. Taken together, our 3C analysis demonstrated that PcG mediated *BX-C* higher order structures do not depend on the Dcr2 pathway.

### Comparison of AGO2-bound sRNAs and Genome-wide PcG Protein Distribution

**Figure 6:**
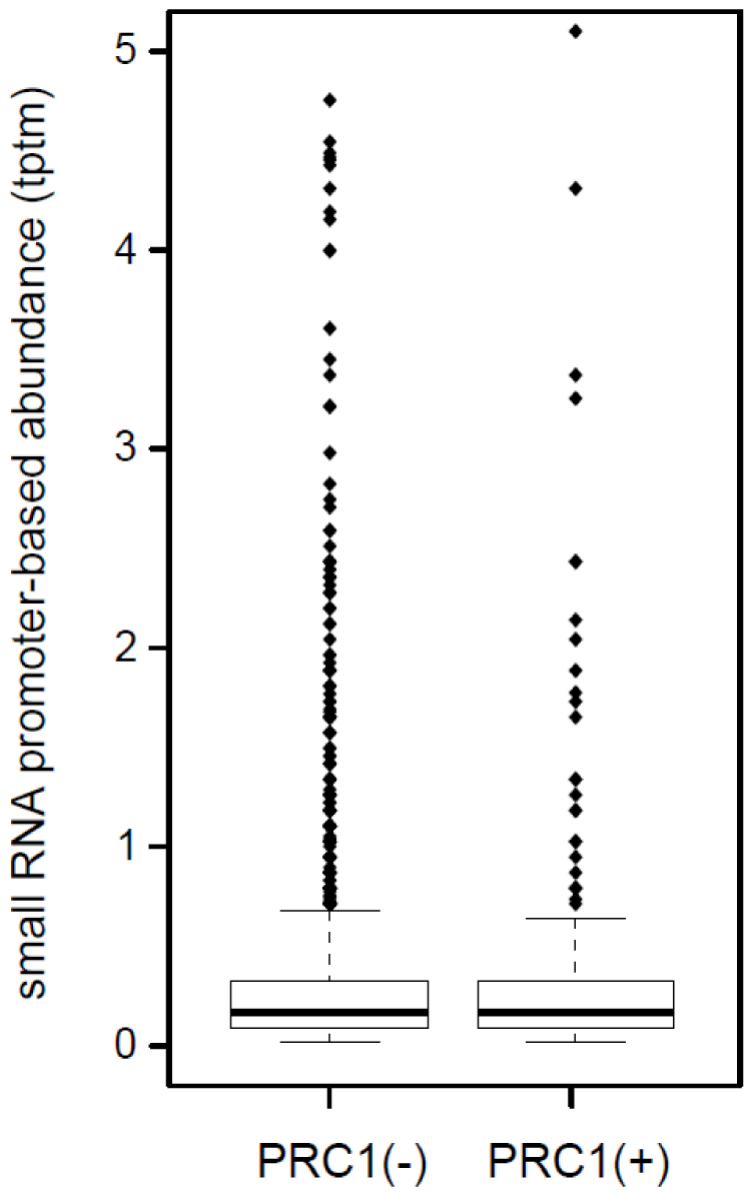
PcG-occupied promoters are not enriched in AGO2-bound proximal promoter-derived small RNA sequences. No significant enrichment in small RNAs observed in PcG-bound promoters relative to promoters without PcG-binding (Kolmogorov–Smirnov test, p-value = 1.0); tag counts are normalized to tags per ten million tags (tptm). Promoter regions were defined as the genome region 50 nucleotides upstream and downstream of the Refseq-defined transcriptional start site, which were linked to PcG occupancy as previously described [50].

PcG proteins are enriched in promoter regions [50]. In addition PRC1 has been shown to preferentially target promoters having a stalled Pol II [50]. As proximal promoter-derived sRNAs associate with the AGO2 protein [20], [51] which has been suggested to control Pol II processivity [20], we investigated a potential overlap between AGO2-bound sRNAs and the genomic regions of PRC1 promoter target regions. To this purpose we analyzed the intersection of PcG ChIP-seq peaks [50] and our AGO2-bound sRNA libraries [20]. We observed no enrichment of sRNAs bound to AGO2 at promoter regions associated with PRC1 proteins relative to promoters lacking PRC1 association (Fig. 6), suggesting there is no link between PRC1 occupancy and RNAi mediated control of Pol II processivity.

## Discussion

Seminal discoveries made in yeast and in plants [18], [52], [53] provided evidence for mechanistic connections between RNAi mechanisms and heterochromatin formation [15], [19], [54]. RNAi pathways appear to play a key role in transcriptional gene silencing and genome integrity [15], [19], [54], [55]. RNA silencing factors are also required for the formation of *Drosophila* pericentric heterochromatin, recruitment of heterochromatin protein 1 (HP1) and silencing of transgenes that are inserted into pericentromeric heterochromatin [21], [56].

Other studies proposed a role for RNAi in PcG mediated gene silencing [38], [39]. A first report showed that RNAi was implicated in the case of repeat-induced gene silencing (analogous to the RNAi-mediated phenomenon of co-suppression described in plants) in which the introduction of array copies of a transgene results in silencing also of the endogenous homologues loci. For this the RNAi factor PIWI was found to be required in combination with components of the PcG to maintain silencing [38]. In a subsequent work, RNAi components were shown to play a role in pairing-sensitive gene silencing of PRE-containing transgenes [39].

The maintenance of H3K27 methylation is central to PcG-dependent silencing processes. Our mass spectrometry analysis in Dcr2 depleted cells did not show any significant increase in the total levels of un-modified H3K27 (Fig. 4), indicating that the action of this protein is not necessary for the histone methyltransferase activity of PRC2. Moreover, we found that depletion of Dcr2 does not affect the silencing of endogenous *BX-C* genes (Fig. 2D) and only mildly derepresses PRE-specific transcripts (Fig. 2B). Grimaud and colleagues [39] reported that defects in Dcr2, AGO1 and PIWI affected transgene silencing and the binding of only some, not all, PcG proteins. We could not detect loss of PcG binding from endogenous PREs. Conversely, our ChIP experiments show that, upon Dcr2 depletion, PC and H3K27m3 levels at PREs are increased rather than lowered (Fig. 3). The reason for this is presently unclear and needs further examination. The observed increase of PC binding (Fig. 3) and total H3K27m3 levels upon Dcr2 depletion (Fig. 3 and 4) might be the result of a compensatory mechanism to prevent activation of *BX-C* genes possibly regulated by PRE-specific non-coding RNAs. Interestingly the increase in PC but not in H3K27m3 positively correlates with transcriptional derepression and Pol II enrichment as indicated by the ChIP analysis on mildly derepressed PREs (Fig. 3). In contrast the non-derepressed *Abd-B* promoter (Fig. S4) only shows an increase in H3K27me3. However, this increase in H3K27m3 does not appear to be specific for PcG bound regions (Fig. 3), and does not accompany increased PC-chromatin interactions. Thus the increase of H3K27me3 seems to be a systemic, perhaps indirect, consequence of Dcr2 depletion.

It has been shown by FISH analysis that in some RNAi mutants, including Dcr2, repressed *HOX* genes lose the ability to interact with each other in the nuclear space [39]. Subsequent work based on an unbiased 3C walk in the repressed *BX-C* performed in RNAse treated cells did not reveal any changes in PRE-mediated higher order structures [33]. In the present work we directly examined the known associations between PcG-bound regulatory elements of the *BX-C* after Dcr2 depletion and found that the higher order structure of the *BX-C* remained largely unaffected (Fig. 5). Notably the frequency of interaction between the *abd-A* promoter and the *Fab-7* PRE was enhanced after Dcr2 depletion (Fig. 5B), although to date, no specific functional role of the *Fab-7* PRE in the regulation of the *abd-A* promoter has been reported. This could be a consequence of the high levels of H3K27m3 and PC at PREs (Fig. 3) causing abnormal interactions in high order structures [49].

An important recent publication showed that AGO2 associates with PREs and that the protein, but not its slicing activity, is required for CTCF-mediated chromatin insulator function, indicating that AGO2 has a RNAi-independent role in chromatin looping [40]. In particular, AGO2 is required for the CTCF-mediated looping between the *Abd-B* promoter A and the *Fab-7/Fab-8* insulators. This finding is not in contrast with our observations showing that DNA looping between PcG binding sites (not insulators) are unaffected by Dcr2 knock-down. Nevertheless, further experiments are needed to precisely describe the functional correlation between insulator-mediated structures, depending on AGO2, and PcG-mediated multi-looped structures.

A clear indication for a Dicer-dependent function in heterochromatin is the production of 21–22 nt RNAs from dsRNA precursors. Despite several efforts, we could not detect RNAi-dependent sRNAs in the *BX-C*. We investigated potential sources of dsRNA at PREs in both active and repressed loci. We found that transcripts from both strands could be detected at PREs (Fig. 1B), but, as previously described, these transcripts appear to correlate with the anti-silencing mode of PREs rather than PcG repression [43]. Further, by comparing published data sets of PcG ChIP-seq peaks [50] and AGO2-bound sRNA libraries [20], we did not identify AGO2-bound sRNAs at PcG target promoters. We have recently shown that Dcr2 and AGO2 proteins are part of the Pol II transcriptional machinery and localize preferentially to euchromatic, transcriptionally active regions [20]. Accordingly, ChIP-seq profiling in *Drosophila* S2 cells revealed that AGO2 is mostly bound to active promoters, overlaps extensively with TrxG proteins and, as shown in a genetic assay, it behaves rather as a *TrxG* gene counteracting PcG function [40]. However the mechanistic link between TrxG-mediated chromatin remodeling, RNAi and control of Pol II processivity has to be addressed with further experiments. Our data clearly show that the RNAi machinery is not involved in PcG mediated silencing at endogenous *HOX* targets and suggest rather a role of RNAi components in opposing PcG repression.

## Methods

### Fly Stocks

Flies were maintained using standard procedures. The *Drosophila melanogaster* strain *Canton-S* was used as source of wild type embryos. The *Fab7ΔPRE^56^* homozygous stock [57] was obtained from F. Karch (Geneva).

### Cell Culture

Schneider 2 (S2) cells were grown in serum free insect medium (HyQ SFX, Hyclone). Schneider 3 (S3) cells were grown in Schneider's *Drosophila* medium (Invitrogen) supplemented with 12,5% fetal bovine serum.

### Protein Depletion via dsRNAs

Production of dsRNAs (400–500 bp long) against Dcr2 was produced by in vitro transcription using T7 polymerase (Mega-Script kit, Ambion). Transcripts were denatured by heating and re-annealed in water by cooling to room temperature. RNAi was performed as described previously [58] and cells were grown for 8–10 days. Primers for production of T7 templates:

GFP-F 5′ACGTAAACGGCCACAAGTTC3′

GFP-R 5′TGCTCAGGTAGTGGTTGTCG3′

DCR2-F 5′GTTCCGCTTTGGTCAACAAT3′

DCR2-R 5′TGATCGTCTTTTCCATGCAG3′

### Antibodies

The antibody against PC was kindly provided by R. Paro [59]; against Dcr2 from Q. Liu [60]. We also used the following commercial antibodies: Dcr2 (Abcam ab4732); Pol II 4H8 (Abcam ab5408), H3K27m3 (Upstate 07–449).

### Primer Sequences used in RT-PCR and ChIP Analyses

Primer pairs are listed in Table S1. The position of PRE-specific primer pairs is given according to the coordinates of the *BX-C* sequence published previously [61]. Accession number #U31961.

Primers for the *Intergenic control region* and the *Abd-B promoter* were described previously [44], [62].

### RT-PCR

Total RNA was extracted using the TRIzol reagent (Invitrogen). Strand specific RT-PCR was performed using the SuperScript One step RT-PCR kit (Invitrogen), Samples, DNase I treated, were incubated with primers complementary to upper or lower strand transcripts in first strand cDNA synthesis reactions. A second primer was added after heat inactivation of the reverse transcriptase at 94°C for 2 min so that both primers were present in the subsequent PCR amplification. RT-PCR scheme: 50°C for 30 min once (cDNA synthesis); 94°C for 2 min once (inactivation of the reverse transcriptase); 94°C for 30 sec, 60°C for 1 min, 68°C for 1 min, 35 times; 68°C for 7 min once (PCR amplification).

For non-strand-specific RT-PCR for PRE transcripts about 1 µg of total RNA from each source was used to reverse transcribe target sequences using the QuantiTect Reverse Transcription kit (Qiagen) according to the manufacturer’s directions. The resulting cDNA was analyzed by PCR using the QuantiTect SYBR Green PCR kit (Qiagen) according to the manufacturer’s instructions. The amplified products were analyzed by electrophoresis. No amplification in RT reactions without addition of the reverse transcriptase confirmed that the starting template was DNA-free RNA. Quantitative real time PCR was performed with the DNA Engine Opticon 2 (MJ). Quantification was normalized to the housekeeping gene *GAPDH1*, and relative expression levels were calculated using the following equation: A = 2^[Ct(ref)-Ct(ref-control)]-[Ct(sample)-Ct(sample-control)]^.

### ChIP Assay

Chromatin was prepared and immunoprecipitated as described previously [44]. Quantitative-PCR was performed in a DNA Engine OPTICON 2 (MJ Research, Bio-Rad) instrument using the QuantiTect SYBR Green PCR Kit (Qiagen) according to manufacturer’s instructions. Relative quantifications were determined from the threshold cycle for amplification using the 2^−ΔCt^ method [63]. The percentage of input shown was corrected by the values of the no antibody control to substract the general background.

### Histone Separation, Processing and MALDI- Mass Spectrometry

About 5–10 pmole of acid extracted histones were separated by SDS-PAGE. Coomassie blue stained bands corresponding to histones H3 and H4 were excised and subjected to chemical modification to derive the free amino groups of lysine residues [47], [64]. Digestions were carried out overnight with sequencing-grade trypsin (Promega,), according to manufacturer’s protocol. Then MALDI spectra were acquired [47], [64]. The resulting spectra were analyzed with Manuelito, an in-house developed software (http://manuelito.sourceforge.net/), and differentially modified peptides were quantified [65], [66]. Briefly, the corresponding peaks were monoisotoped and integrated. The total cluster area of the different isoforms of one peptide was taken as 100% and the contribution of each isoform to the total determined from the integrated area under each peak.

### Chromosome Conformation Capture (3C)

The 3C assay was performed essentially as described previously [33]. Primer sequences are available on request.

### RNAse Protection Assay

The RNase protection assay was performed using the mirVana miRNA detection kit (Ambion) according to the manufacturer’s instructions with minor modifications. Briefly, a single stranded α-^32^P-UTP-labeled RNA molecule was hybridized with 20 µg of RNA. The RNA preparations are enriched for molecules smaller than 200 nucleotides (mirVana miRNA isolation kit, Ambion). Overnight hybridization was carried out at 44°C and digestion with RNAseA/RNAseT1 (1/50 dilution) was performed at 37°C for 1 hour. Protected fragments were loaded on 16% acrylamide/8 M urea gels and visualized using a Phosphorimager (Typhoon Amersham-Bioscience). Single-stranded RNA molecules were used as molecular weight markers (Decade marker Ambion). The radio-labeled ssRNA probe was prepared by *in vitro* transcription (Maxi-Script kit, Ambion) of the DNA amplicons containing T7 promoters. To increase the specific-activity of the RNA probes the unlabeled UTP was not included in the transcription reaction. The full-length RNA probe was gel-purified using an 8% acrylamide/8 M urea gel and eluted overnight at 37°C. Primer sequences for the production of the DNA amplicons containing T7 promoters are available on request. The miRNA-bantam target DNA template was amplified with T7 and T3 primers from pBS-miRNA-bantam-target [45] kindly provided by M. Siomi and H. Siomi.

### Computational Analysis of AGO2-bound sRNAs

Basic processing of sRNA libraries is as described previously [20] with this analysis including only the sRNA libraries sequenced under standard conditions. To understand the distribution of sRNA sequences over *BX-C*-sized genomic regions in *Drosophila*, randomized genome sequences of equal length to *BX-C* were extracted from the drosophila dm3 genome assembly [67] and total sRNA counts over the genomic interval were tabulated with a custom perl script calling shuffleBed and other utilities from the bedtools utilities suite [68] while excluding the *BX-C* region from the randomized selections. The promoter regions of genes (defined as +/−50 nt around the RefSeq transcriptional start site) linked to the previously-determined PcG ChIP-seq peaks [50] were inspected for overlap with AGO2-bound sRNA sequences. Statistical significance of AGO2-bound sRNA overlap between groups of genes with or without PcG presence was determined with the Kolmogorov–Smirnov (k–s) test as implemented by the R language and environment for statistical computing. All large scale comparisons were performed using the samtools software package [69] and bedtools utilities suite [68].

## Supporting Information

Figure S1 **The** ***BX-C*** **is not enriched in AGO2-bound small RNAs**. Histogram with each bin containing the number of trials (y-axis) with the labeled number of sRNA counts (x-axis) out of a total of 10,000 trials. Each trial sums the total number of sRNA sequences found along randomly- selected genomic regions of equivalent length to the *BX-C* region (∼340 kb). The placement of the actual sRNA count for the *BX-C* region in the histogram is denoted with an arrow on the x-axis.
(TIF)

Figure S2 **Homologous small-RNAs are not found at the PRE regions of the** ***BX-C*** **.** (**A–D**) RNAse protection analysis. ^32^P-UTP radiolabeled RNA probes from the indicated regions have been incubated with an equivalent amount of yeast RNA (control) or *Drosophila* small-RNAs (shorter than 200 nt) from S2 cells, S3 cells or embryos (0–18 h after egg laying; wild type or carrying a deletion of the *Fab-7* PRE ). Only the portion of the probe paired with complementary RNA molecules will be protected from RNAse cleavage (arrow head). The protected fragments produced in presence of yeast RNA are considered background. (**D**) As comparison we detected the presence of the miRNA bantam. E: wild type embryos; E ΔFab7: embryos carrying a deletion of the *Fab-7* PRE.
(TIF)

Figure S3 **Dcr2-depletion does not cause transcriptional activation of homeotic genes.** (**A–B**) Quantitative RT-PCR analysis of core PRE transcripts. (**A**) The samples analyzed were mock-treated S2 cells (control without dsRNA) or S2 cells treated with Dcr2 dsRNA, (**B**) S3 cells and embryos (0–18 h after egg laying). (**C**) Quantitative RT-PCR of the *BX-C* homeotic genes. The samples analyzed were mock-treated S2 cells (control without dsRNA) or S2 cells treated with Dcr2 dsRNA. The expression levels are shown as fraction of *GAPDH* transcripts. The results shown are from three independent experiments; error bars show the standard deviation.
(TIF)

Figure S4 **Dcr2-depletion does not alter PC and Pol II occupancy at the** ***Abd-B*** **promoter.** Crosslinked chromatin from mock treated S2 cells (control transfection without the dsRNA) or S2 cells treated with Dcr2 dsRNA was immunoprecipitated with the antibodies indicated below the bars (H3K27m3, PC, Pol II). The immunoprecipitated DNA was analyzed by quantitative PCR with primers specific for the *Abd-B promoter* [44]. Protein binding is expressed as a percentage of input minus the background signal. The results shown are from three independent experiments; error bars show the standard deviation.
(TIF)

Table S1 **Primer sequences used in RT-PCR and ChIP analyses.** The position of PRE-specific primer pairs is given according to the coordinates of the *BX-C* sequence published previously [61]. Accession number #U31961. Primers “b” generate shorter amplicons that have been used for qPCR analysis. Primers for the *Intergenic control region* and the *Abd-B promoter* were described previously [44], [62].
(PDF)
