# Supplementary figures and images for: RNA-Interference Components Are Dispensable for Transcriptional Silencing of the Drosophila Bithorax-Complex
Source: PLoS One. 2013 Jun 13;8(6):e65740. doi: 10.1371/journal.pone.0065740 (PMC3681981; doi:10.1371/journal.pone.0065740)

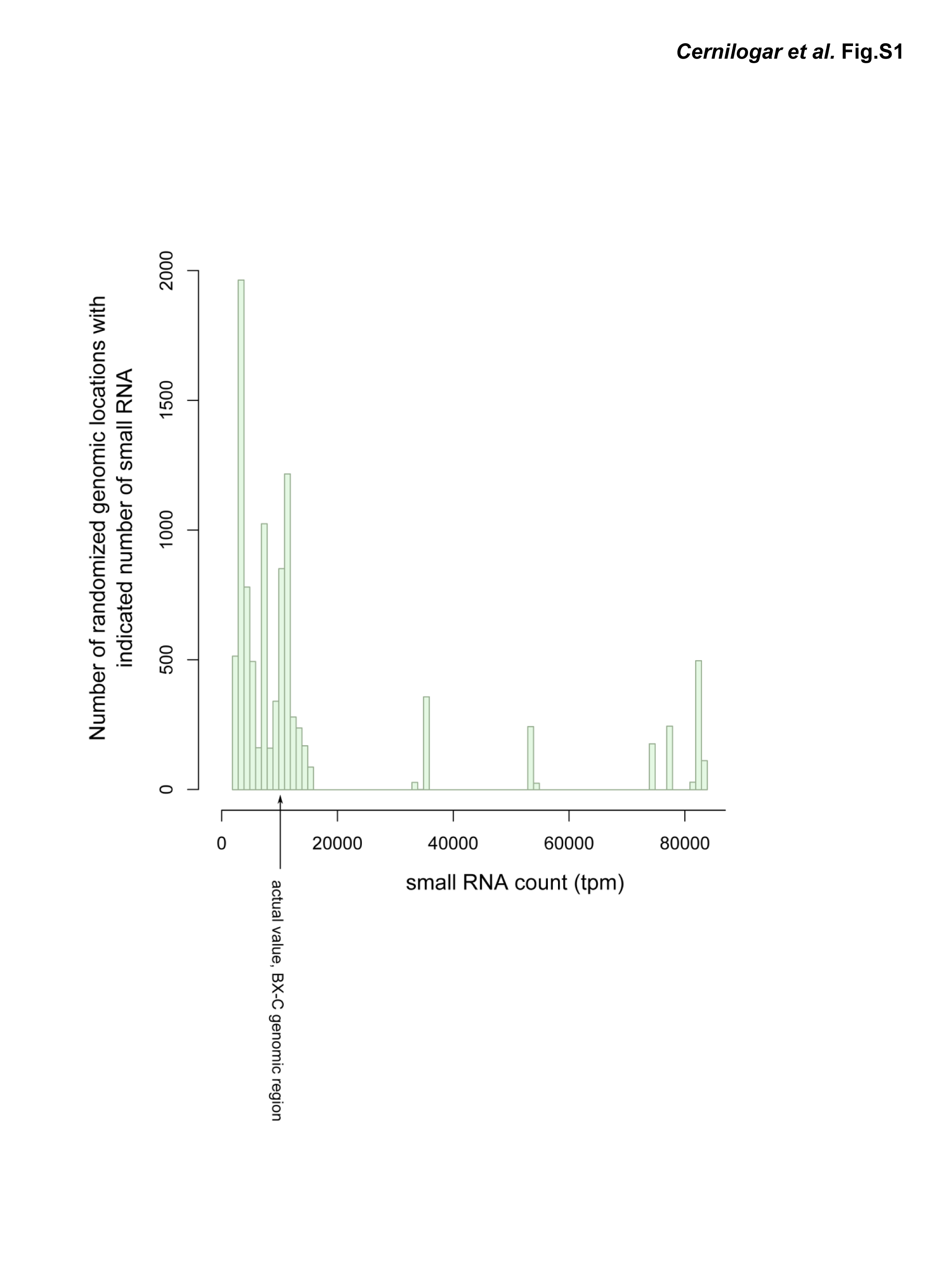

Supplement: Figure S1 — The BX-C is not enriched in AGO2-bound small RNAs. Histogram with each bin containing the number of trials (y-axis) with the labeled number of sRNA counts (x-axis) out of a total of 10,000 trials. Each trial sums the total number of sRNA sequences found along randomly- selected genomic regions of equivalent length to the BX-C region (∼340 kb). The placement of the actual sRNA count for the BX-C region in the histogram is denoted with an arrow on the x-axis. (TIF) [file pone.0065740.s001.tif]

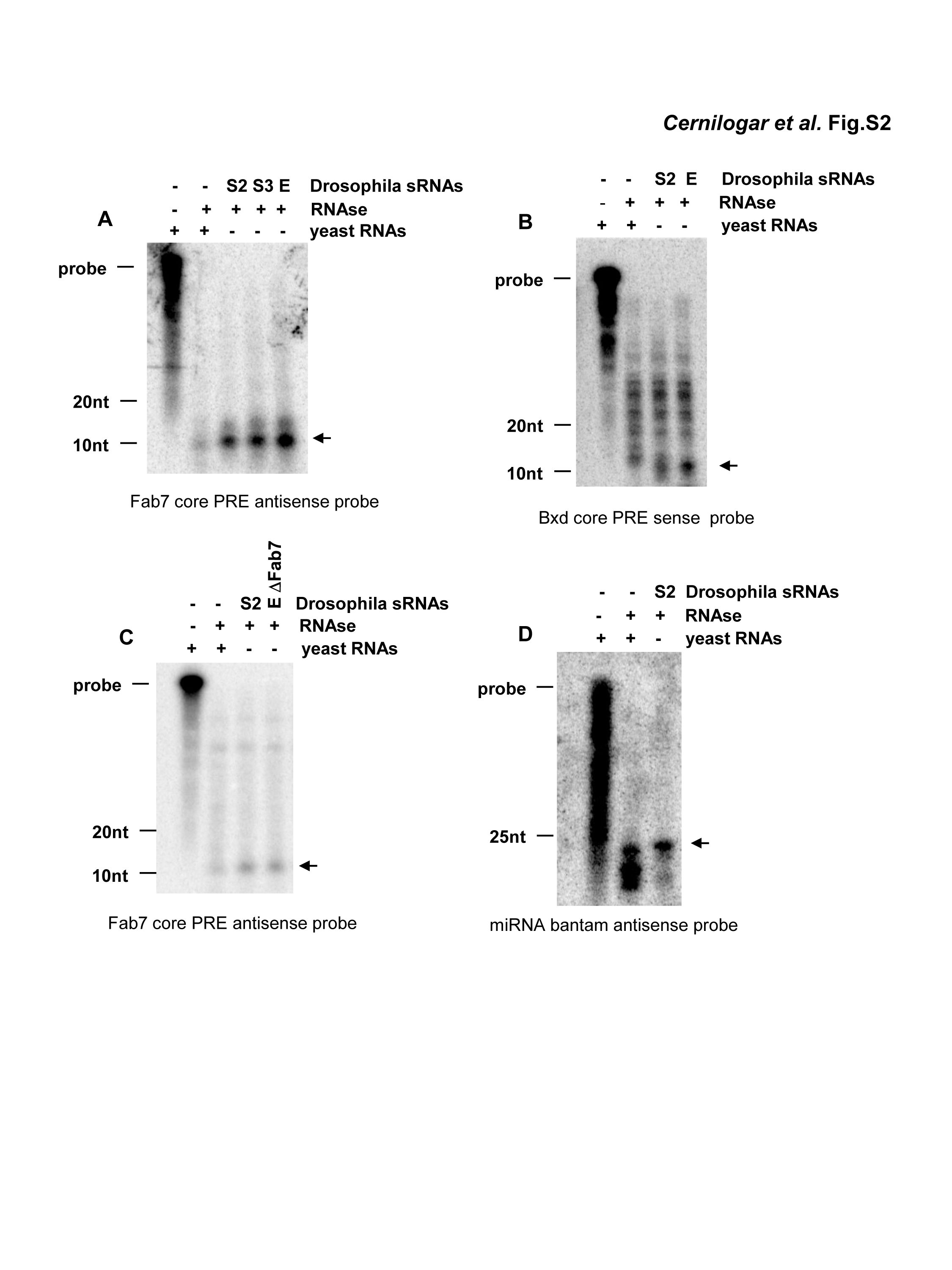

Supplement: Figure S2 — Homologous small-RNAs are not found at the PRE regions of the BX-C . (A–D) RNAse protection analysis. 32P-UTP radiolabeled RNA probes from the indicated regions have been incubated with an equivalent amount of yeast RNA (control) or Drosophila small-RNAs (shorter than 200 nt) from S2 cells, S3 cells or embryos (0–18 h after egg laying; wild type or carrying a deletion of the Fab-7 PRE ). Only the portion of the probe paired with complementary RNA molecules will be protected from RNAse cleavage (arrow head). The protected fragments produced in presence of yeast RNA are considered background. (D) As comparison we detected the presence of the miRNA bantam. E: wild type embryos; E ΔFab7: embryos carrying a deletion of the Fab-7 PRE. (TIF) [file pone.0065740.s002.tif]

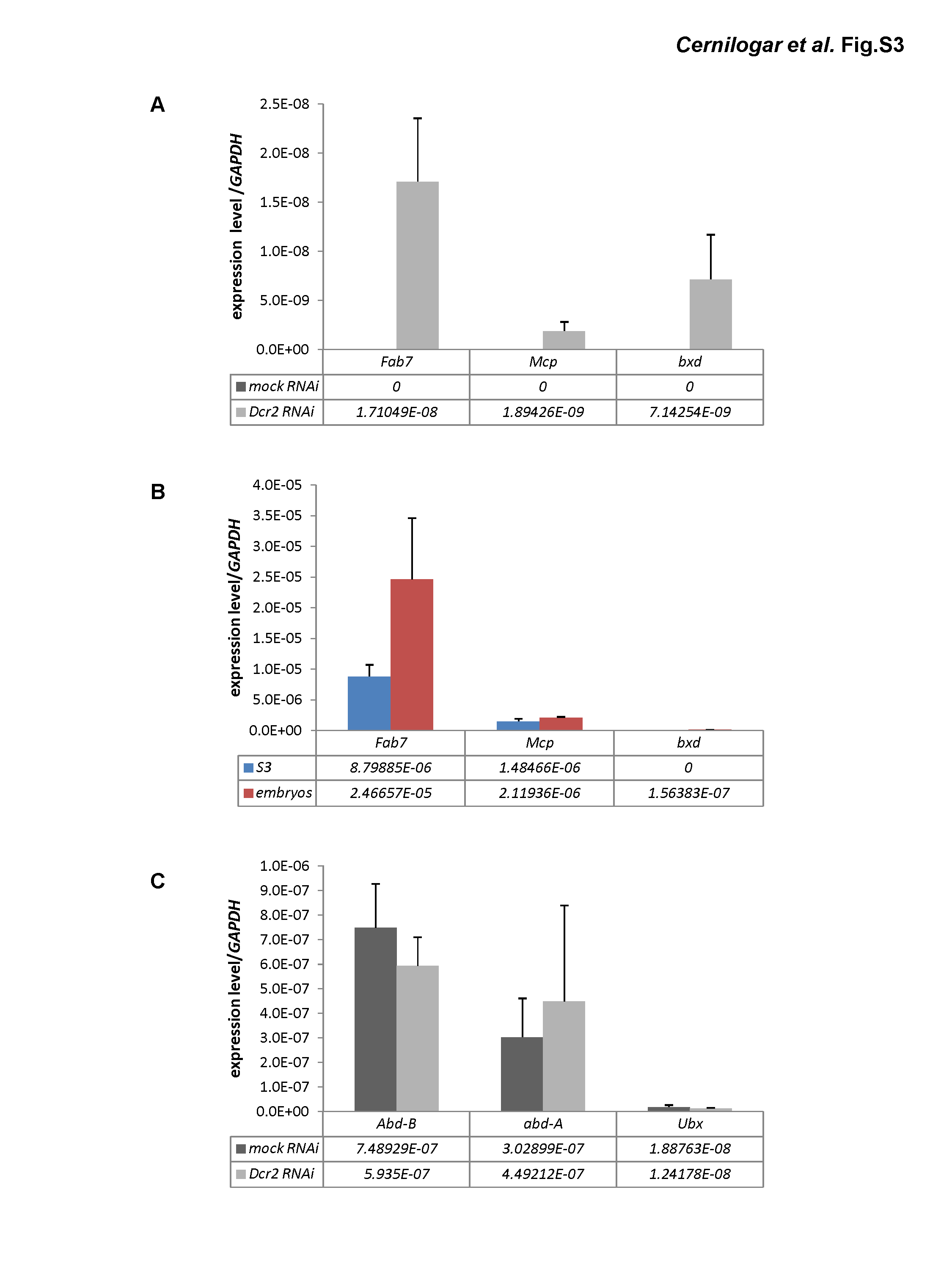

Supplement: Figure S3 — Dcr2-depletion does not cause transcriptional activation of homeotic genes. (A–B) Quantitative RT-PCR analysis of core PRE transcripts. (A) The samples analyzed were mock-treated S2 cells (control without dsRNA) or S2 cells treated with Dcr2 dsRNA, (B) S3 cells and embryos (0–18 h after egg laying). (C) Quantitative RT-PCR of the BX-C homeotic genes. The samples analyzed were mock-treated S2 cells (control without dsRNA) or S2 cells treated with Dcr2 dsRNA. The expression levels are shown as fraction of GAPDH transcripts. The results shown are from three independent experiments; error bars show the standard deviation. (TIF) [file pone.0065740.s003.tif]

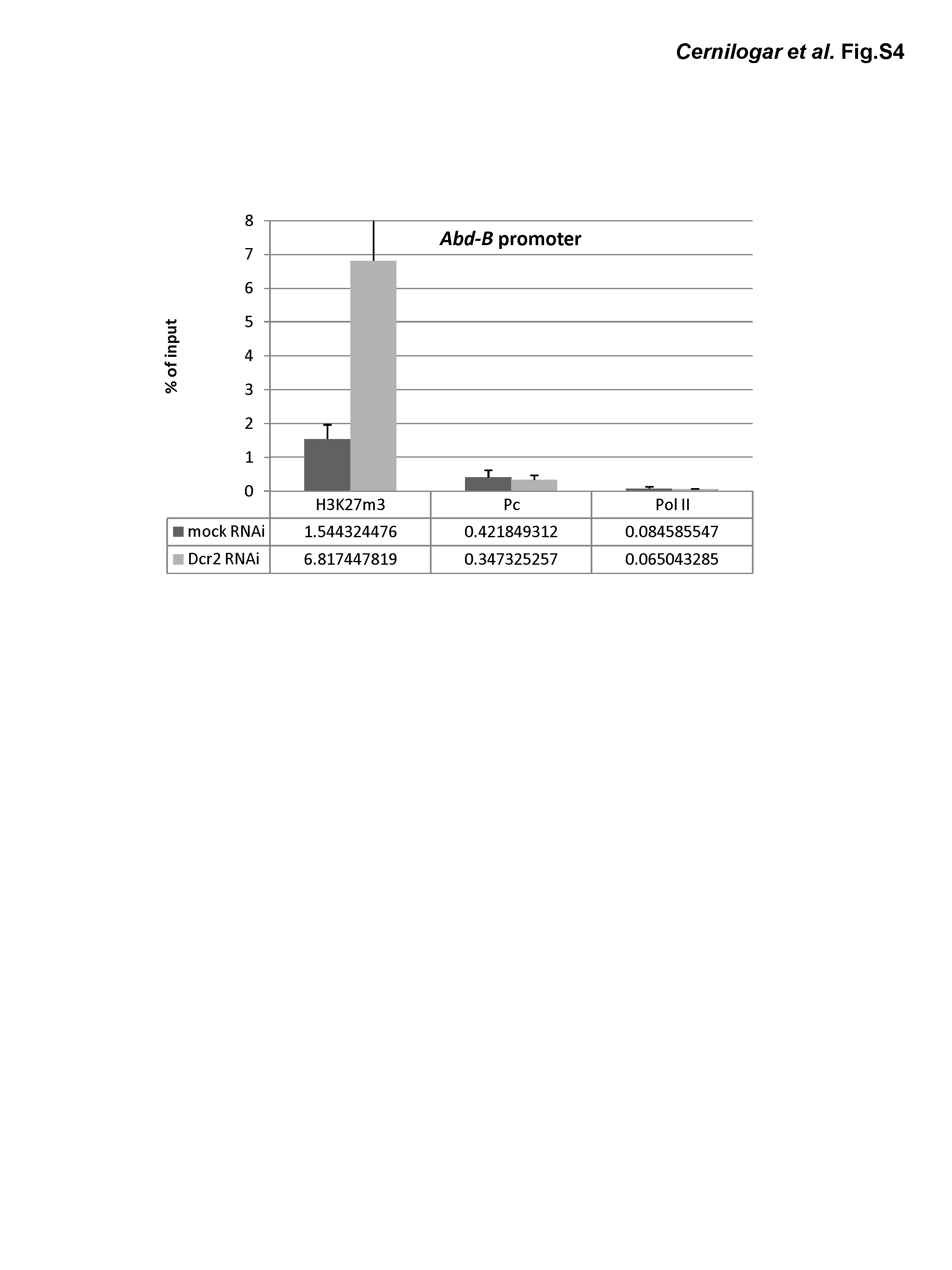

Supplement: Figure S4 — Dcr2-depletion does not alter PC and Pol II occupancy at the Abd-B promoter. Crosslinked chromatin from mock treated S2 cells (control transfection without the dsRNA) or S2 cells treated with Dcr2 dsRNA was immunoprecipitated with the antibodies indicated below the bars (H3K27m3, PC, Pol II). The immunoprecipitated DNA was analyzed by quantitative PCR with primers specific for the Abd-B promoter [44]. Protein binding is expressed as a percentage of input minus the background signal. The results shown are from three independent experiments; error bars show the standard deviation. (TIF) [file pone.0065740.s004.tif]
