# Supplementary material for: RNA-Interference Components Are Dispensable for Transcriptional Silencing of the Drosophila Bithorax-Complex
Source: PLoS One. 2013 Jun 13;8(6):e65740. doi: 10.1371/journal.pone.0065740 (PMC3681981; doi:10.1371/journal.pone.0065740)
Supplement: Table S1 — Primer sequences used in RT-PCR and ChIP analyses. The position of PRE-specific primer pairs is given according to the coordinates of the BX-C sequence published previously [61]. Accession number #U31961. Primers “b” generate shorter amplicons that have been used for qPCR analysis. Primers for the Intergenic control region and the Abd-B promoter were described previously [44], [62]. (PDF) [file pone.0065740.s005.pdf]

Table S1.

Primer sequences used in RT-PCR and ChIP analyses

The position of PRE-specific primer pairs is given according to the coordinates of the *BX-C* sequence published previously [61]. Accession number #U31961.

PRE-specific primer pairs		Sequence (5´-3´)	Position
Fab-7-f9	forward	ggttcggtaagaggtctac	83167-83185
	reverse	gaacttcacaacagacgacg	83621-83639
Fab-7-f9b	forward	gaaaatgcccaacaaaatgc	83361-83380
	reverse	cgctgtctcgcctcttcttc	83473-83492
Mcp-m6	forward	acactcacacatgagacag	112305-112323
	reverse	ttgacggcaatactcaagg	112772-112790
Mcp-m6b	forward	cccagagtctttggacttgc	112460-112479
	reverse	cataaagcgaggaacgaagg	112632-112651
bxd-b2	forward	agtgcggtgataaggtcc	218802-218819
	reverse	gacaaaccattcagattcag	219389-219408
bxd-b2b	forward	gcactcaaaatccgaaaatg	219041-219060
	reverse	cacgtcagacttggaatagc	219168-219187
**Other primer pairs**		**Sequence (5´-3´)**	
AGO1	forward	tgagcatcatcttccactgc	
	reverse	ctatggtgcatgcctatagc	
GAPDH1	forward	aagggaatcctgggctacac	
	reverse	accgaactcgttgtcgtacc	
Abd-B	forward	gctagtccagcgattggaag	
	reverse	gtcggttggtcacacatcag	
Abd-a	forward	caaatacaacgcaacccgagac	
	reverse	agcgatcgtgttgctgctg	
Ubx	forward	agtgtcagcggcggcaac	
	reverse	agtctggtagaagtgagcccg	

Primers "b" generate shorter amplicons that have been used for qPCR analysis.

Primers for the *Intergenic control region* and the *Abd-B promoter* were described previously [44,62].
